# A Case Report: Diagnosis of *Mycobacterium kansasii* Infection by tNGS in a Patient With Parkinson’s Disease

**DOI:** 10.1155/crdi/5084676

**Published:** 2026-10-28

**Authors:** Shunmei Gao, Fangfang Deng, Gegang Wang

**Affiliations:** ^1^ Department of Infectious Diseases Chongqing Wulong People’s Hospital Wulong Chongqing 408500 China; ^2^ Infection Diagnosis Center Sichuan Kingmed Center for Clinical Laboratory Co., Ltd Chengdu 610052 Sichuan China

**Keywords:** lung cancer, *Mycobacterium kansasii*, Parkinson’s disease, targeted next-generation sequencing

## Abstract

Infections caused by nontuberculous mycobacteria (NTM), especially *Mycobacterium kansasii,* are characterized by nonspecific clinical manifestations. Moreover, their radiological features frequently overlap with those of both tuberculosis (TB) infections and lung cancer, which significantly complicates the differential diagnosis. We present a case of a patient with Parkinson’s disease (PD) who was admitted with cough and sputum production and dyspnea. Chest computed tomography (CT) revealed atelectasis, multiple solid nodules, osteolytic lesions in multiple ribs, and pleural effusion. The initial diagnostic evaluation raised the suspicion of a lung cancer with multiple bone metastases. While acid‐fast staining (AFS) of biopsy tissue was positive, polymerase chain reaction (PCR) for TB was negative. Ultimately, targeted next‐generation sequencing (tNGS) of sputum detected *M. kansasii*, considering the diagnosis of pulmonary *M. kansasii* infection. This case highlights the need to consider *M. kansasii* infection in atypical imaging findings and the utility of tNGS for diagnosis when conventional tests are inconclusive.

## 1. Introduction

No‐tuberculous mycobacteria (NTM) are a group of opportunistic pathogens widely distributed in the environment. They can infect multiple parts of the human body; the lung is the most frequently infected organ [1]. *Mycobacterium kansasii* is the sixth most common NTM globally. A meta‐analysis demonstrates that it accounts for 9.4% of the NTM isolates, while the proportion of *M. kansasii* among NTM pulmonary infection varies regionally, ranging from 3% to 70% [2]. Pulmonary *M. kansasii* infection frequently presents with cough, chest pain, and dyspnea, and its clinical manifestations resemble those of tuberculosis (TB) [3]. When pulmonary *M. kansasii* infection presents with multiple bone destructions, the imaging findings closely resemble those of bone metastases from lung cancer [4]. There have been previous reports on misdiagnosis of pulmonary *M. kansasii* infection as lung cancer [5–7]. However, cases of *M. kansasii* infection accompanied by multiple bone destructions misdiagnosed as a lung cancer in patients with Parkinson’s disease (PD) are exceedingly rare.

The conventional microbiological tests for NTM include bacterial culture, acid‐fast staining (AFS), and polymerase chain reaction (PCR), and they have notable limitations in clinical practice. Targeted next‐generation sequencing (tNGS) is a key advancement in sequencing technology. It offers multiple advantages, including high throughput, improved sensitivity, and short detection time [8]. In common lower respiratory tract infections, blinded qualitative tNGS outperformed conventional microbiological testing (CMT) (sensitivity: 78.5% vs. 58.7% and specificity: 76.6% vs. 74.4%). Model‐guided tNGS achieved the highest diagnostic accuracy, with a sensitivity of 82.4% and a specificity of 85.0%. By contrast, CMT combined with clinical manifestations only reached a sensitivity of 68.8% and a specificity of 72.0% [9]. For the diagnosis of NTM infection, previous studies demonstrated that the detection sensitivity of tNGS for NTM is 42.6%, surpassing the 36.5% sensitivity of PCR [10]. These studies reveal that tNGS enable rapid pathogen identification, facilitating clinical diagnosis and treatment. Here, we describe a patient with PD complicated by *M. kansasii* infection, emphasizing the utility of tNGS in resolving the diagnostic challenges posed by its atypical manifestations.

## 2. Case Presentation

A 60‐year‐old man had a 3 years history of Grade 3 hypertension and PD. He presented with a 20‐day history of cough, sputum production, dyspnea, and persistent right‐sided chest pain. He had undergone clinical evaluation at another hospital. Bronchoalveolar lavage culture grew *Staphylococcus aureus*. Transbronchial biopsy demonstrated acute and chronic inflammation with reactive lymphoid hyperplasia, granuloma, and abscess formation. Whole‐body bone scintigraphy revealed multiple foci of increased radiotracer uptake in the mandible, clavicles, multiple ribs, sternum, pelvis, and humeri. These results raised high suspicion for lung cancer with multiple bone metastases. The patient was transferred to our hospital for personal reasons.

On admission, his temperature was 36.3°C, with a heart rate of 78 beats per minute, a respiratory rate of 23 breaths per minute, and a blood pressure of 133/70 mmHg. Physical examination revealed decreased breath sounds in the right upper and bilateral lower lung fields, coarse breath sounds bilaterally, and scattered moist rale. Chest computed tomography (CT) demonstrated stenosis and occlusion of the right upper lobe bronchus with associated atelectasis, large bilateral pleural effusions, multiple solid pulmonary nodules, and osteolytic lesions involving multiple ribs and thoracolumbar vertebrae. Pleural fluid was pale yellow and clear, with a positive Rivalta test. Pleural fluid analysis showed the following: white blood cell count 1335 × 10^6^ cells/L, adenosine deaminase (ADA) 3.00 U/L, lactate dehydrogenase (LDH) 93.00 U/L, and total protein 26.10 g/L. Based on a combination of the patient’s clinical manifestations, prior imaging, and bone scintigraphy findings, the leading differential diagnosis was lung cancer with multiple bone metastases. Concurrent pulmonary infection was also considered.

On hospital Day 1, carcinoembryonic antigen (CEA) and squamous cell carcinoma antigen (SCC) were within normal limits. Screening tests for hepatitis B virus (HBV), hepatitis C virus (HCV), syphilis, and human immunodeficiency virus (HIV) were negative. Antibiotic therapy was switched to amoxicillin–clavulanate (1.2 g every 8 h), with concurrent symptomatic supportive care.

On hospital Day 6, the patient developed intermittent high‐grade fever (peak 39.4°C), and his cough and dyspnea persisted without improvement. To further characterize the pulmonary lesion, lung biopsy was performed. Histopathological examination of the biopsy tissue revealed alveolar epithelial hyperplasia and granuloma formation, suggestive of interstitial pneumonia. AFS of the biopsy tissue was positive, suggestive of mycobacterial infection.

On hospital Day 7, TB‐PCR of the biopsy tissue was negative. A definitive diagnosis of TB infection could not be established at the time.

On hospital Day 11, AFS of three consecutive sputum smears returned positive. Based on the histopathological result and sputum AFS, empiric anti‐tuberculosis (anti‐TB) therapy was initiated with an oral fixed dose combination of isoniazid, rifampicin, pyrazinamide, and ethambutol 2.7 g once daily.

On hospital Day 12, *M. kansasii* was detected in sputum by tNGS (Table 1). The diagnosis was revised to pulmonary *M. kansasii* infection. The empiric anti‐TB regimen was promptly discontinued, and a four‐drug antinontuberculous mycobacterial (anti‐NTM) regimen was initiated. It consisted of oral azithromycin 0.5 g once daily, moxifloxacin 0.4 g once daily, ethambutol 1.0 g once daily, and rifampicin 0.6 g once daily.

**TABLE 1:** Results of tNGS in sputum of patient.

Sample type	Genus name	Species name	Normalized reads per million (RPM)	Estimated pathogen concentration (copies/mL)
Sputum	*Mycobacterium*	*Mycobacterium kansasii*	12,898	> 1.0E + 6

On hospital Day 15, lymph node ultrasonography identified bilateral cervical, axillary, and inguinal lymphadenopathy. Excisional biopsy of the left inguinal lymph node was performed to rule out lymphoma. Histopathological examination returned chronic lymphadenitis, and immunohistochemistry (IHC) excluded lymphoma.

Following targeted anti‐NTM therapy, the patient’s fever resolved gradually, with improvement in cough and dyspnea. On hospital Day 18, the patient was discharged at his request on a four‐drug oral anti‐NTM regimen to complete treatment at a local facility. One month after discharge, the patient showed marked improvement in cough and dyspnea, with notably increased exercise tolerance. There was no recurrence of high grade fever or chest pain. Compared with admission, CT demonstrated partial resolution of the pulmonary lesions (Figure 1). The timeline of this case report is provided in Table 2.

**FIGURE 1:**
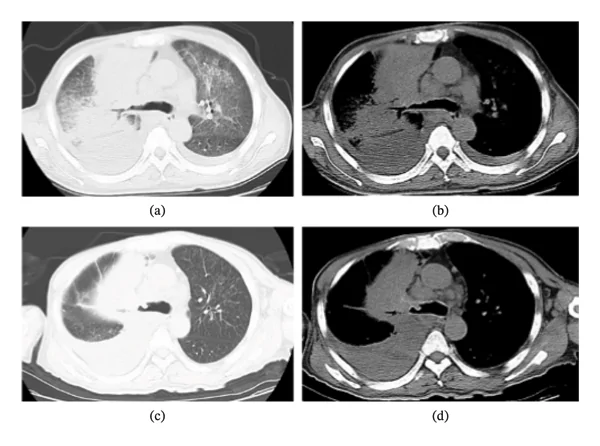
(a, b) Imaging of the lung window and mediastinal window at admission. (c, d) Imaging of the lung window and mediastinal window at discharge.

**TABLE 2:** Timeline.

Day	Event
0	Cough, sputum production, dyspnea, and persistent right‐sided chest pain; empiric anti‐infective therapy
1	Negative CEA and SCC; negative serology for HBV, HCV, syphilis, and HIV; antibiotic switch to amoxicillin–clavulanate
6	Intermittent fever (peak 39.4°C); unrelieved cough and dyspnea; granulomas on lung biopsy, AFS of lung biopsy tissue (+)
7	Negative TB‐PCR of lung biopsy tissue
11	Three sputum smears AFS (+); empiric oral anti‐TB HRZE therapy
12	*M. kansasii* detected by sputum tNGS; anti‐TB discontinued, four‐drug anti‐NTM regimen initiated (moxifloxacin, azithromycin, ethambutol, rifampicin)
13	Whole‐body lymph node ultrasonography: bilateral cervical, axillary, and inguinal lymphadenopathy
15	Excisional biopsy of left inguinal lymph node
18	Discharged on four‐drug oral anti‐NTM regimen (moxifloxacin, azithromycin, ethambutol, rifampicin)
19	Left inguinal lymph node biopsy: chronic lymphadenitis; lymphoma ruled out by IHC
48	Improved exercise tolerance; resolved cough and dyspnea; no recurrent fever and chest pain; CT showing lesion improvement

*Note:* +: positive. CEA, carcinoembryonic antigen; SCC, squamous cell carcinoma antigen; HBV, hepatitis B virus; HCV, hepatitis C virus; TB, tuberculosis; HRZE, isoniazid, rifampicin, pyrazinamide, ethambutol; IHC, immunohistochemistry.

Abbreviations: AFS, acid‐fast staining; CT, computed tomography; HIV, human immunodeficiency virus; NTM, nontuberculous mycobacteria; PCR, polymerase chain reaction; tNGS, targeted next‐generation sequencing.

## 3. Discussion

We report a case of pulmonary *M. kansasii* infection in a patient with PD. The patient presented with multiple pulmonary nodules and multiple bone destructions. Imaging findings were highly suggestive of lung cancer with multiple bone metastases. Conventional diagnostic methods failed to identify the pathogen, which was ultimately detected by tNGS of the sputum specimen.

As described in previous reports, the clinical symptoms and imaging of pulmonary *M. kansasii* disease are often resemble those of pulmonary TB and lung cancer [3, 4]. *M. kansasii* mainly infects individuals with underlying basic pulmonary comorbidities (such as bronchiectasis and chronic obstructive pulmonary disease) or immunosuppressive conditions (such as HIV infection and long‐term immunosuppressive therapy) [11]. In contrast, the patient lacked all the aforementioned classic risk factors for NTM infection but presented with right upper lung bronchial stenosis, multiple pulmonary nodules, bilateral pleural effusion, and multiple bone destructions. Initially, it was highly suspected to be a lung cancer with multiple bone metastases. This highly atypical presentation highlights the diagnostic challenge and increases the risk of misdiagnosis.

Conventional microbiological tests for NTM have obvious limitations. Mycobacterial culture is the traditional gold standard, but NTM culture takes several weeks, which may delay treatment; AFS has low specificity and cannot distinguish NTM from TB; PCR detection targets specific gene fragments (such as *16S rRNA* and *hsp65*), but there are primer mismatches among different NTM species [12, 13]. tNGS can identify pathogens directly from clinical specimens within 12–24 h through specific primer amplification combined with high‐throughput sequencing [14]. In the NTM pulmonary disease, the sensitivity and specificity of tNGS were 100% and 97.5%, respectively [15], proving it to be a valuable auxiliary diagnostic tool in the NTM infections. In this case, AFS of the lung biopsy tissue was positive and TB‐PCR was negative, but tNGS detected *M. kansasii* directly from the sputum specimen. This case illustrates the complementary diagnostic value of tNGS when conventional test fails.

PD is the second most common neurodegenerative disorder worldwide, and it has two core respiratory pathophysiological alterations. First, dysphagia arises from dysfunction of the dopaminergic neural network and the accumulation of Lewy bodies and α‐synuclein. It reduces swallowing efficiency and may lead to secretion retention and aspiration. Second, the accumulation of α‐synuclein in the medulla oblongata weakens respiratory muscle, while reduction in neural drive impairs cough reflex. Together, they lead to disruption of airway protection [16]. This patient had no classic risk factors for NTM infection (such as HIV and long‐term immunosuppression) and has only a 3‐year history of PD. We speculate that PD‐related impairment of airway defense mechanisms may have reduced clearance of inhaled environmental mycobacteria, and this may be a potential factor. However, this remains a mechanistic hypothesis.

This case has several notable features. First, we searched PubMed for case reports published in the past 10 years using the keywords “*M. kansasii*” and “lung cancer.” The search identified two cases initially misdiagnosed as lung cancer without bone involvement [6, 7]. Another case occurred in an immunocompromised patient and was misdiagnosed as lung cancer with bone destruction [5]. Unlike those cases, our patient had no known immunodeficiency‐related risk factors, such as HIV infection or long‐term immunosuppressive therapy. The only notable medical history was PD. To date, no report has described a patient with PD who had pulmonary *M. kansasii* infection, multiple bone destructions, and imaging findings mimicking lung cancer metastases. This case expands the clinical and imaging spectrum of *M. kansasii* infection. Second, in this case, AFS was positive, while TB‐PCR was negative. Conventional microbiological tests failed to identify the pathogen, but tNGS clearly demonstrated its diagnostic superiority over conventional microbiological tests. Third, this case puts forward the hypothesis that PD‐related airway defense impairment may increase susceptibility to NTM infection. This provides a new insight into potential risk factors for this disease.

Nevertheless, this case also has several limitations. First, while tNGS offers better sensitivity and specificity than conventional methods, it is not the gold standard but rather an auxiliary diagnostic tool. In this case, culture confirmation of *M. kansasii* was not available. Although the patient showed a favorable clinical response to targeted anti‐NTM therapy, which supported the diagnosis, the lack of culture confirmation remains a major limitation of this case. Second, informed consent for vertebral tissue sampling could not be obtained. Consequently, we have no direct pathological or microbiological evidence to confirm that bone destruction resulted from *M. kansasii* infection. We cannot entirely rule out the possibility that bone destruction represented a separate unrelated process. Third, as a single case, the hypothesis of a relationship between PD and NTM susceptibility cannot be applied to clinical practice. Further epidemiological and mechanistic studies are required for validation.

The diagnostic challenge in this case provides a useful reference for the evaluation of similar conditions. When a patient presents with pulmonary nodules and multiple bone destructions, with imaging findings highly suggestive of malignancy, if repeated pathological examinations show no malignant cells and AFS is positive while TB‐PCR is negative, NTM infection should be considered in the differential diagnosis. tNGS of deep respiratory specimen can rapidly identify the pathogen. This approach may help avoid misdiagnosis and unnecessary anticancer or anti‐TB therapy, thereby improving patient outcomes.

## 4. Conclusion

This case emphasizes the clinical diagnostic challenges of *M. kansasii* infection. These challenges are particularly notable in patients with atypical manifestations and carry important clinical‐reference implications. (1) *M. kansasii* infection should be considered in patients with chronic respiratory symptoms, pulmonary nodules, and granulomatous pathology, even without classic NTM risk factors; (2) tNGS can effectively overcome the limitations of conventional diagnostic methods (such as culture and PCR),and provide a rapid and accurate tool for identifying *M. kansasii*; and (3) PD may be a potential risk factor for NTM infection.

## Author Contributions

Shunmei Gao contributed to conception of the case and manuscript preparation. Fangfang Deng carried out manuscript drafting and subsequent revisions. Gegang Wang undertook manuscript review and research coordination.

## Funding

No funding or sponsorship was received for this research or the publication of this article.

## Consent

The patient allowed personal data processing, and informed consent was obtained from the patient participants included in the study.

## Conflicts of Interest

The authors declare no conflicts of interest.

## Supporting Information

Additional supporting information can be found online in the Supporting Information section.

## Supporting information


**Supporting Information** CARE‐checklist.

## Data Availability

The data that support the findings of this study are available from the corresponding author upon reasonable request.
