# Supplementary material for: A Case Report: Diagnosis of Mycobacterium kansasii Infection by tNGS in a Patient With Parkinson’s Disease
Source: Case Rep Infect Dis. 2026 Oct 28;2026:5084676. doi: 10.1155/crdi/5084676 (PMC13717076; doi:10.1155/crdi/5084676)
Supplement: Supplementary file 1 [file CRDI-2026-5084676-s001.pdf]

CARE case report guidelines

# CARE Checklist of information to include when writing a case report

(cc) BY-NC-ND

| Topic | Item | Checklist item description | Reported on Line |
|---|---|---|---|
| **Title** | 1 | The diagnosis or intervention of primary focus followed by the words "case report" | Line1-2/Page1 |
| **Key Words** | 2 | 2 to 5 key words that identify diagnoses or interventions in this case report, including "case report" | Line23-24/Page1 |
| **Abstract (no references)** | 3a | Introduction: What is unique about this case and what does it add to the scientific literature? | Line11-14/Page1 |
| | 3b | Main symptoms and/or important clinical findings | Line14-17/PAge1 |
| | 3c | The main diagnoses, therapeutic interventions, and outcomes | Line17-20/Page1 |
| | 3d | Conclusion—What is the main "take-away" lesson(s) from this case? | Line20-22/Page1 |
| **Introduction** | 4 | One or two paragraphs summarizing why this case is unique (**may include references**) | Line48-50/Page2 |
| **Patient Information** | 5a | De-identified patient specific information. | Line52/Page2 |
| | 5b | Primary concerns and symptoms of the patient. | Line52-53/Page2 |
| | 5c | Medical, family, and psycho-social history including relevant genetic information | Line52/Page2 |
| | 5d | Relevant past interventions with outcomes | Line53-59/Page2 |
| **Clinical Findings** | 6 | Describe significant physical examination (PE) and important clinical findings. | Line60-63/Page2 |
| **Timeline** | 7 | Historical and current information from this episode of care organized as a timeline | Line107/Page4 |
| **Diagnostic Assessment** | 8a | Diagnostic testing (such as PE, laboratory testing, imaging, surveys). | Line63-87/Page3 |
| | 8b | Diagnostic challenges (such as access to testing, financial, or cultural) | Line58/Page2 |
| | 8c | Diagnosis (including other diagnoses considered) | Line87-88/Page3 |
| | 8d | Prognosis (such as staging in oncology) where applicable | Line96-101/Page4 |
| **Therapeutic Intervention** | 9a | Types of therapeutic intervention (such as pharmacologic, surgical, preventive, self-care) | Line86-91/Page3-4 |
| | 9b | Administration of therapeutic intervention (such as dosage, strength, duration) | Line86-91/Page3-4 |
| | 9c | Changes in therapeutic intervention (with rationale) | Line87/Page3 |
| **Follow-up and Outcomes** | 10a | Clinician and patient-assessed outcomes (if available) | N/A |
| | 10b | Important follow-up diagnostic and other test results | N/A |
| | 10c | Intervention adherence and tolerability (How was this assessed?) | N/A |
| | 10d | Adverse and unanticipated events | N/A |
| **Discussion** | 11a | A scientific discussion of the strengths AND limitations associated with this case report | Line150-174/Page7 |
| | 11b | Discussion of the relevant medical literature **with references**. | Line119-153/Page6-7;Line206-250/Page8-9 |
| | 11c | The scientific rationale for any conclusions (including assessment of possible causes) | Line115-149/Page5-7 |
| | 11d | The primary "take-away" lessons of this case report (without references) in a one paragraph conclusion | Line175-189/Page7-8 |
| **Patient Perspective** | 12 | The patient should share their perspective in one to two paragraphs on the treatment(s) they received | N/A |
| **Informed Consent** | 13 | Did the patient give informed consent? Please provide if requested | **Yes** ☑ **No** ☐ |
